# Patient Involvement in Health Technology Assessments: Lessons for EU Joint Clinical Assessments

**DOI:** 10.3390/jmahp13030038

**Published:** 2025-07-28

**Authors:** Anne-Pierre Pickaert

**Affiliations:** Acute Leukemia Advocates Network (ALAN), CH-3000 Bern, Switzerland; anne-pierre@care4access.fr

**Keywords:** health technology assessments, HTA, joint clinical assessments, patient involvement

## Abstract

Patient involvement in health technology assessment (HTA) processes is increasingly recognized as pivotal for informed, equitable, and patient-relevant health care decision-making. With the implementation of Joint Scientific Consultations (JSCs) and Joint Clinical Assessments (JCAs) under Regulation (EU) 2021/2282, the European Union has a unique opportunity to design harmonized mechanisms that reflect best practices from established HTA systems. This article, drawing on the Acute Leukemia Advocates Network (ALAN)’s comparative analysis of HTA practices across seven countries (Canada, England, Scotland, France, Germany, Spain, and Italy), examines how current patient involvement processes can inform the JCA framework. It identifies opportunities to replicate effective practices and proposes strategies to embed patient voices meaningfully into the JCA process. By prioritizing robust and inclusive patient involvement, the EU can establish a global benchmark for impactful and consistent HTA processes. By leveraging lessons from international HTA systems and prioritizing clear frameworks, early involvement, and capacity building, the EU can set a global standard for meaningful patient participation in HTA processes. ALAN is an independent global network of patient organizations dedicated to improving outcomes for patients with acute leukemia.

## 1. Introduction

Health Technology Assessment (HTA) is a multidisciplinary process that uses explicit methods to determine the value of a health technology at different points in its lifecycle. Its purpose is to inform decision-making that promotes equitable, efficient, and high-quality health systems [1]. Regulation (EU) 2021/2282, in force since January 2025, seeks to enhance access to innovative health technologies, ensure the efficient use of resources, and strengthen the quality of HTA across the EU [2]. The regulation focuses on the clinical aspects of HTA, including joint clinical assessments (JCAs) of new medicines and devices and joint scientific consultations (JSCs) to provide early advice to technology developers [3].

Under the regulation, Member States are required to give due regard to JCAs, but retain authority over drawing the final conclusions on the overall value of a new health technology, as well as pricing and reimbursement decisions [3].

Patient involvement in HTA is increasingly recognized as essential for improving the relevance and quality of health care decision-making. Patients provide unique insights into lived experiences, treatment priorities, and real-world challenges, complementing clinical and economic assessments. Including patient perspectives enhances the legitimacy and transparency of decision-making processes, ensuring that recommendations align with patient needs. However, patient involvement remains inconsistent across Europe, with varying levels of integration depending on the HTA agency [4]. These inconsistencies present a major challenge for JCAs, which aim to establish a harmonized approach to HTA across EU Member States. Without clear, structured mechanisms for patient involvement, there is a risk that JCAs will lack the patient-relevant insights necessary to guide effective decision-making at the European level.

This article builds on ALAN’s comparative report on patient involvement in HTA [5], which analyzed patient involvement practices across seven countries. While the ALAN’s comparative report focused on describing existing practices, identifying opportunities for patient involvement in national HTA bodies and exploring its impact on decision-making, this paper extends that analysis by focusing on lessons relevant for JCAs and offers concrete recommendations for integrating patient perspectives into the EU HTA framework. By highlighting both best practices and persistent gaps, this article aims to provide practical guidance for policymakers, HTA bodies, and patient organizations on establishing a consistent, meaningful approach to patient involvement in JCAs.

## 2. Materials, Methods, and Study Limitations

### 2.1. Study Objectives

This article synthesizes key findings from a comparative report on patient involvement in HTA published in 2024 by ALAN [5]. The report examines opportunities for patient engagement and its impact on medicine reimbursement decisions across seven jurisdictions:

- Canada: Canadian Agency for Drugs and Technologies in Health (CADTH), now Canada’s Drug Agency (CDA-AMC);
- England: National Institute for Health and Care Excellence (NICE);
- Scotland: Scottish Medicines Consortium (SMC);
- France: French National Authority for Health (HAS);
- Germany: Institute for Quality and Efficiency in Health Care (IQWiG) and Federal Joint Committee (G-BA);
- Italy: Italian Medicines Agency (AIFA);
- Spain: Spanish Agency for Medicines and Medical Devices (AEMPS).

This article builds on the key findings from the report by focusing specifically on the relevance of its findings to EU Joint Clinical Assessments (JCAs). It aims to carry out the following:

- Showcase the diversity of patient involvement practices across HTA agencies and explore how cross-agency learning can inspire innovation and best practices in patient engagement at both national and EU levels.
- Provide actionable recommendations for integrating meaningful and consistent patient engagement within the EU JCA framework.

### 2.2. Study Design and Approach

The research followed a two-phase desk research methodology, combining targeted literature searches and document analysis.

Phase one focused on English-language sources, reviewing official HTA agency websites, policy documents, annual reports, and procedural guidelines. Additional sources were identified through Google, Google Scholar, and PubMed searches to identify peer-reviewed studies, grey literature, consultant reports, and news articles published between January 2020 and April 2023. The research emphasized explicit policies on patient involvement, procedural framework, and real-world examples of how patient input has been integrated into HTA decision-making. The English-language literature search yielded 26 peer-reviewed articles and 20 grey literature sources on patient involvement in HTA, along with 13 peer-reviewed articles and three grey literature sources focusing on the impact of patients and patient group involvement on decision-making. Agency-specific documents included 15 documents from CADTH (now CDA-AMC), 25 from NICE, 15 from SMC, and 1 from Health Improvement Scotland.

Recognizing that important HTA documentation is available in national languages, the second phase of the review expanded to non-English sources. This included an analysis of HTA websites and grey literature in French, German, Italian, and Spanish, covering publications from 1 January 2016 to 1 November 2023 (Italian and Spanish), and from 1 January 2016 to 13 February 2024 (French and German). The review included official HTA agency materials, government reports, and additional grey literature, focusing on identifying differences in patient involvement approaches across jurisdictions.

Desk research in this phase yielded the following results: two French-language grey literature sources and thirty-six documents from HAS; one peer-reviewed article in German, two documents from G-BA, and eighteen from IQWiG; two Italian-language grey literature sources and twenty-one documents from AIFA; and five Spanish-language grey literature sources, along with two documents from AEMPS, and three from the Spanish Ministry of Health.

### 2.3. Study Limitations

ALAN’s comparative report has several limitations. First, despite systematic desk research, it is possible that some relevant webpages, policy documents, or grey literature sources were missed. Second, the scope of the analysis was limited to patients and patient group involvement in the HTA of medicines for reimbursement decisions; it did not explore patient roles in HTA agency governance structures or regulatory processes, which may offer additional insights. Third, the English and non-English sources were reviewed by two different researchers, which may introduce human error or unconscious bias in document selection and interpretation. Fourth, the review did not include the literature published after May 2023 (for CADTH, NICE, and SMC) or after March 2024 (for HAS, G-BA, IQWiG, AIFA, and AEMPS), meaning more recent developments may not be reflected.

## 3. Comparative Analysis of Patient Involvement in HTA

### 3.1. Diversity in Terminology

A critical finding is the inconsistent terminology used across jurisdictions, with terms such as “patient representation”, “stakeholder engagement”, and “public participation” often used interchangeably, causing confusion. Translation differences further complicate understanding. For example, HAS refers to “patient contribution”, while IQWiG and G-BA use terms like “patient representation” or “participation of the affected persons”, reflecting varying levels of involvement.

The term “patients” itself covers diverse stakeholders, including individual patients, carers, patient advocates, organization representatives, and patient experts. While agencies like CDA-AMC, HAS, G-BA, and AEMPS primarily engage patient groups, NICE and IQWiG also support individual patient involvement, especially when no groups exist.

None of the reviewed HTA agencies involve individuals with lived experience in decision-making roles. CDA-AMC, NICE, HAS, G-BA, and SMC include representatives who may be patients with conditions other than the one under review or other lay representatives. The inconsistent use of terms such as lay representatives, patient representatives, consumers, users, and public partners—sometimes within the same agency—further complicates patient participation. These inconsistencies risk creating barriers to effective, harmonized patient involvement across Member States.

### 3.2. Variability in Information and Support Provided

Terms like “patient”, “community”, “member of the public”, and “health care users” are used across CDA-AMC, HAS, IQWiG, and SMC websites. However, AIFA and AEMPS lack clear signposting for patients or patient group involvement, suggesting a lower prioritization of such engagement.

Agencies with a longer history of patient involvement, including CDA-AMC, NICE, HAS, IQWiG, and SMC, generally have clear policies and in-house teams supporting patient engagement (except IQWiG). In contrast, AIFA and AEMPS lack publicly available policies or dedicated support teams, with minimal methodological guidance on patient roles in HTA processes. According to the Office of Health Economics [6], this reflects a stronger focus on affordability and budget impact over patient value in these systems.

This indicates that agencies with established patient engagement practices tend to formalize their approach, with clear policies and dedicated resources to support meaningful involvement.

### 3.3. Differences in the Stages of Patient Involvement

Patient involvement in HTA processes spans multiple stages, providing essential contributions that enhance meaningful and transparent decision-making. Key stages include the following:

- Early Advice: Patients provide insights into unmet needs and treatment priorities, shaping early assessment objectives. NICE, CDA-AMC, and HAS integrate patient input during this stage, corresponding to the Joint Scientific Consultation phase at the EU level;
- Scoping: Patient contributions refine research questions and assessment objectives, aligning evaluations with real-world concerns. NICE and IQWiG lead in this stage;
- Pre- and post-draft recommendations: Patients review findings, fostering transparency. NICE and SMC offer structured processes for patient feedback;
- Appeals: Processes such as NICE’s appeal procedure and SMC’s PACE meetings allow patients to contest decisions and influence outcomes, particularly for rare or life-threatening conditions.

Table 1 outlines how HTA agencies approach these stages. While the stages are modelled on NICE’s comprehensive process, they serve as a generic framework for comparing practices across agencies.

### 3.4. Importance of Resourcing and Capacity Building

Effective patient engagement in HTA requires substantial investment in financial, human, and technical resources. Robust organizational capacity is essential for ensuring equitable and meaningful participation.

- Leading examples: Agencies like NICE and CDA-AMC excel in capacity building through training programmes, guidance materials, and patient advisory committees. They also allocate resources to support and compensation, ensuring continued engagement.
- Challenges: AIFA and AEMPS, by contrast, rely on less structured, ad hoc approaches, limiting their ability to sustain meaningful engagement.

### 3.5. Measuring Impact

Despite the increasing recognition of patient involvement as a critical component of decision-making, systematic evaluation remains a significant gap.

CDA-AMC and NICE have begun tracking patient contributions, measuring the number, quality, and influence of submissions on recommendations. However, these efforts remain limited to partial indicators, and most agencies lack comprehensive and systematic frameworks to assess how patient input meaningfully affects HTA processes and outcomes. Such frameworks are essential to move beyond simple metrics and ensure that patient involvement is evaluated in terms of both its qualitative and quantitative impact on decision-making. Without clear methodologies for assessing this impact, patient contributions risk being undervalued or inconsistently integrated into final recommendations, limiting the effectiveness and legitimacy of the process.

Research using both qualitative and quantitative methods has yielded mixed results, indicating that patient involvement is often seen as beneficial but reliant on sufficient resourcing. In some cases, patient input has a minimal impact, particularly when clinical data quality is weak, or cost-effectiveness thresholds remain unmet.

### 3.6. Overview of Differing Practices Across Jurisdictions

Patient involvement practices vary widely across countries and regions, influenced by cultural, institutional, and policy contexts. This makes it more important to ensure that patient involvement is actively enabled and supported through appropriate structures and resources, so that meaningful engagement is possible in all settings. The primary method for patient input is pre-recommendation written submissions, where patients share treatment experiences and highlight unmet needs and priorities. However, the availability of guidance materials and submission templates differs (see Table 2).

#### 3.6.1. Leaders in Patient Involvement

NICE (England), SMC (Scotland), and CDA-AMC (Canada) demonstrate best practices with structured guidelines, advisory panels, and transparent feedback mechanisms. These agencies exemplify a transparent and inclusive approach to patient involvement, emphasizing both written input and direct participation.

NICE offers more opportunities for patients and patient group involvement throughout the HTA process than any of the other agencies looked at. It integrates patient involvement at all stages of HTA, including the following:

- Scoping: NICE is the only agency with a formal scoping phase, where patients help refine research questions and identify key outcomes through written submissions or workshops;
- Pre-draft recommendation input: Written submissions from patient groups are collected, and patient experts may attend committee meetings to clarify evidence and respond to questions. Observers can attend public committee sessions. For certain evaluations, only written inputs are used, without direct patient group participation;
- Post-draft recommendation input: NICE allows 28 days for patient groups and others to provide written comments on draft recommendations;
- Decision-making: Patient representatives participate in deliberations and have the same voting rights as other decision-making committee members;
- Feedback Mechanisms: NICE provides detailed explanations of how patient input influenced decision outcomes;
- Appeals: NICE is the only agency offering a formal appeal process for patient groups.

SMC is renowned for its Patient and Clinician Engagement (PACE) meetings, which provide an additional opportunity for patient involvement in the case of end of life, orphan, and ultra-orphan treatments. Key features include the following:

- Pre-recommendation input: Patient groups submit written inputs, guided by templates. Representatives may attend meetings where a summary of their contributions is presented, and they can clarify points when needed. Meetings are held publicly, and a list of participating patient groups is published;
- Post-draft recommendation input: If the New Drugs Committee (NDC) does not recommend a treatment, a PACE meeting may take place for end-of-life, orphan, and ultra-orphan treatments, allowing patients and caregivers to share their experiences directly;
- Decision-making: Patient representatives participate in decision-making with full voting rights.

CDA-AMC exemplifies a structured, transparent, and inclusive approach to patient involvement. Patients are engaged early and consistently, supported by dedicated resources. Mechanisms include the following:

- Pre-draft recommendation input: CDA-AMC emphasizes written submissions only from patient groups, supported by templates and guidance. Submissions are published in full and summarized in draft recommendations. No direct participation in committee meetings is offered;
- Post-draft recommendation input: Patient groups have 10 days to provide feedback on draft recommendations, focusing on whether their input was adequately considered;
- Decision-making: Patient representatives participate in CDA-AMC’s deliberations and have the same voting rights as other decision-making committee members.

#### 3.6.2. Patient Involvement Supporters

HAS (France) and IQWiG and G-BA (Germany) offer moderate patient participation opportunities, but with fewer comprehensive practices compared to the leading agencies.

HAS involves patients primarily during the early advice and pre-recommendation stages:

- Early advice: Individual patients can share experiences of living with the condition and their expectations for future treatments. Additionally, they answer specific questions from the assessment team to define the population, a comparator, and outcome measures;
- Pre-draft recommendation input: Patient groups complete a standardized template, covering disease burden, current treatment experiences, and expectations for new technologies. Patient input is published alongside final recommendations. In 2022, a total of 107 contributions were submitted, contributing to 254 HTA decisions made during that year [7];
- Decision-making: Patient representatives participate in deliberations with voting rights.

The G-BA and IQWiG prioritize patient group involvement as mandated by law:

- Pre-draft recommendation input: G-BA and IQWiG primarily gather patient input through written submissions using standardized templates that cover disease experiences, therapy gaps and expectations from new treatments.
- Decision-making: Patient representatives may participate in decision-making committees, but without voting rights.

While these agencies support patient involvement, their frameworks are less comprehensive, particularly in the areas of direct patient engagement and influence on final recommendations. 

#### 3.6.3. Limited Partners in Patient Involvement

AIFA and AEMPS provide limited engagement opportunities and minimal publicly available information on patient involvement:

- Post-draft recommendation input: AEMPS shares drafts of the HTA report with patient organizations, allowing a 15-day review period before public release.
- Challenges: Both agencies’ limited involvement appears to reflect a lack of political commitment and prioritization of patient input.

## 4. From Evidence to Action: Discussion and Recommendations for Patient Involvement in JCAs

### 4.1. Clarify Terminology and Establish a Predictable Framework for Patient Involvement

A clear, consistent roadmap for patient involvement is essential for meaningful and harmonized participation across the EU. Standardizing terminology and participation processes will promote transparency, reduce confusion, and ensure patients and their representatives are adequately prepared to contribute effectively to JCAs.

#### 4.1.1. Defining “Patients” and Inclusive Involvement

While the European Commission acknowledges the importance of involving both individual patients and patient organizations, the definition should explicitly include carers, patient advocates, and patient experts to capture the full spectrum of stakeholders. Standardization should also address translation challenges to maintain consistency across Member States.

#### 4.1.2. Role of Patient Organizations and Practical Tools

Patient organizations offer a collective, evidence-based perspective often grounded in datasets, such as surveys, interviews, and focus groups. They are well positioned to identify expert patients, especially for rare diseases or conditions with low survival rates. These organizations can also provide training and support for inexperienced participants, as demonstrated by the G-BA in Germany, where patient groups receive compensation for their assistance [8,9].

To support consistency and effectiveness, the European Commission should develop multilingual resources, including the following:

- Clear definitions and standardized terminology;
- Clarified roles and responsibilities for patient contributors;
- Step-by-step participation guidance, including recruitment and feedback mechanisms;
- Accessible materials in multiple languages and formats.

### 4.2. Ensure Early and Consistent Patient Involvement Throughout the JSC and JCA Processes

Patient organizations, health care professionals, non-governmental organizations in the field of health, and other experts should be informed about JCA and JSC timelines as early as possible to facilitate meaningful participation [10]. Ideally, notification should occur as soon as the marketing authorization application is submitted to the European Commission, so that there is sufficient time to identify patients with lived experience and train relevant patients.

Delaying patient involvement until after the scope or draft reports are finalized risks tokenistic participation. Patients should be engaged from the preparatory phase, contributing to defining the assessment scope, drafting reports, and shaping the PICO (Population, Intervention, Comparison, Outcome) framework, especially patient-relevant outcomes and endpoints [9].

A predictable framework for effective participation should include the following:

- Clear timelines for when patient contributions will be requested throughout the JSC and JCA processes;
- Clarifications on the type of information requested and the format for submitting insights;
- Opportunities for both oral and written contributions, aligning with EMA and leading HTA agency practices;
- Plain language summaries of the technologies under assessment [9].

### 4.3. Allocate Resources and Build Capacity for Effective Participation

The European Commission must provide financial and structural support for effective and equitable patient involvement.

- Provide at least two weeks of advance notice for patients to prepare meaningful input;
- Establish dedicated patient engagement teams within HTA agencies [9];
- Expand access to initiatives such as EUCAPA and HTA4Patients, while promoting additional multilingual training to improve patient preparedness;
- Allocate funding for travel, stipends, and honoraria to prevent the exclusion of individuals from lower socio-economic backgrounds [10].

### 4.4. Document, Evaluate, and Report Patient Contributions

Providing feedback not only strengthens transparency, but also reassures patients that their contributions are valued, enhancing their motivation for future participation [9].

- Maintain detailed records of patient input and its influence on decisions;
- Provide structured feedback explaining how patient insights were used;
- Regularly publish engagement summaries, modelled after CDA-AMC reporting, with plain language versions in all EU languages.

To complement this, the EU should establish metrics to assess patient impact:

- Quantitative data (e.g., number of patient submissions per stage).
- Qualitative data (e.g., influence on outcomes and recommendations).

### 4.5. Leverage Digital Tools for Inclusive Participation

Digital tools can enhance accessibility and reach, particularly for marginalized and geographically distant groups.

- Develop a central multilingual EU portal for patient involvement resources, timelines, and feedback.
- Offer virtual participation options at all stages—from early advice to appeals—to reduce financial and geographic barriers.

### 4.6. Promote Cross-Agency Learning and Best Practice Sharing

The European Commission should foster collaboration and knowledge-sharing across Member States:

- Create a central repository of European and international best practices and case studies.
- Host annual EU-level workshops for continuous learning, open to a wide range of stakeholders, including patients, patient organizations, HTA bodies, clinicians, policymakers, and other relevant stakeholders.

## 5. Conclusions

Patient involvement is essential to ensuring that HTAs are equitable, effective, and reflective of patients’ needs. The transition to JCAs under Regulation (EU) 2021/2282 presents a transformative opportunity to integrate meaningful and consistent patient engagement into the EU’s health care decision-making framework.

By leveraging lessons from international HTA systems and prioritizing clear frameworks, early involvement, and capacity building, the EU can set a global standard for meaningful patient participation in HTA processes.

## Figures and Tables

**Table 1:** Approaches to Involvement in the Stages of HTA.

	Canada CDA-AMC	England NICE	France HAS	Germany IQWiG	Germany G-BA	Italy AIFA	Scotland SMC	Spain AEMPS
**Scoping**								
Written submission	N/A	✓ (28 days)	N/A	✓ (-)	N/A	N/A	N/A	N/A
Workshop/Meeting	N/A	✓	N/A	✓ ^a^	N/A	N/A	N/A	N/A
**Pre-recommendation**								
Written submission	✓ (7 weeks)	✓ (12 weeks)	✓ (45 days ^b^)	✓ (15 working days) ^c^	✘ ^d^	✘	✓ (6–8 weeks)	✓ (15 days) ^e^
Template(s)	✓	✓	✓	✓	✘	✘	✓	✘
Guidance	✓	✓	✓	✘	✘	✘	✓	✘
Participation in committee meeting	✘	✓ ^f^	✘ ^g^	✘	✓	✘	✓	✘
**After draft recommendation**								
Written submission	✓ (10 days)	✓ (28 days)	✘	✓ (-)	✘	✘	N/A ^h^	✓
Template	✓	✓	✘	✘	✘	✘	N/A ^h^	✘
Guidance	✓	✘	✘	✘	✘	✘	N/A ^h^	✘
Participation in committee meeting	✓	✘	✘	✘	✘	✘	✓ ^h^	✘
**Appeal**								
Written appeal	✘	✓	✘	✘	✘	✘	N/A ^i^	✓
Oral appeal	✘	✓	✘	✘	✘	✘	N/A ^i^	✘

Notes: ✓ = materials found on the HTA website and/or in other sources. ✘ = not found on the HTA website and/or in other sources. All sources used for each HTA agency are set out in Appendix 2 of the ALAN’s comparative report [5]. N/A = not applicable, as no sources reviewed identified scoping phases at these agencies. (-) details not found for timelines. (^a^) Source for this is not available in English. In Germany, once the G-BA commissions IQWiG to conduct an assessment, there is no formal scoping phase as part of early benefit assessment process. According to feedback from IQWiG, patient representatives at the G-BA Pharmaceuticals subcommittee can, however, participate in discussions on the PICO criteria (Patient, Intervention, Comparator, and Outcomes), which are agreed for the dossier assessment. (^b^) Delays vary between a couple of weeks to a maximum of 45 days, depending on the workload of HAS and the level of urgency. (^c^) IQWiG has been using a template since 2011 (IQWiG, August 2021) that the G-BA’s Coordination Committee for Patient Representation sends to patient organizations it identifies as relevant to contribute to the early benefit assessment process. (^d^) According to feedback from the G-BA, there is no patient involvement in the assessment of orphan medicines. G-BA has the exclusive responsibility for assessing and appraising orphan medicines. (^e^) Although it is not explicitly mentioned on AEMPS’s webpage, patient organizations, together with scientific societies and autonomous communities, can make comments on the draft document during the public consultation period (15 days). (^f^) Although not necessarily for all HTAs, as NICE determines when to invite patients and patient groups. There may also be opportunities for involvement in technical engagement, where it is conducted. (^g^) French patient organizations do not have the option to present their own written contribution during meetings of the Transparency Commission (Commission de la Transparence, CT). The CT oversees the HTA process and has twenty-nine members, three of which are consumer and patient representatives. Instead, their written contributions are presented to all members of the CT by one of its consumer and patient representatives for discussion. (^h^) There can be a Patient and Clinician Engagement (PACE) meeting if New Drugs Committee does not recommend the drug for reimbursement and the company requests the PACE process held for treatments given at the end of life and orphan and ultra-orphan treatments. Conflict of interest statements are typically required. (^i^) SMC feedback that there is no draft recommendation stage and no opportunity for appeal.

**Table 2:** Features of patients and patient group involvement.

	Canada CDA-AMC	England NICE	France HAS	Germany IQWiG	Germany G-BA	Italy AIFA	Scotland SMC	Spain AEMPS
**Organizational approach to involvement**								
Statement/policy on involvement	✓	✓	✓ ^a^	✓	✓	✘	✓	✘
In-house team to support involvement	✓	✓	✓ ^a^	✘	✓	✘	✓	✓ ^a^
Opportunity for involvement in early advice	✓	✓	✓	N/A	✓	✘	✘	✘
Who can be involved: Patients	✘ ^b^	✓	✘	✓	✘	✓ ^c^	✓ ^d^	✘
Who can be involved: Patient groups	✓	✓	✓	✓	✓	✘	✓	✓ ^a^
Members of decision-making group	✓ ^e^	✓ ^f^	✓ ^g^	✘	✓	✘	✓ ^h^	✘
Voting rights	✓	✓	✓ ^g^	✘	✘	✘	✓ ^h^	✘
**Education and/or training**								
Patients	✘	✓	✓ ^a^	✘	✘	✘	✓ ^j^	✘
Patient groups	✓	✓	✓ ^a^	✘ ^i^	✓	✘	✓	✘
**Review of submission**								
Patients	✘	✘	✘	✘	✘	✘	✘	✘
Patient groups	✘	✘	✘	✘	✘	✘	✓	✓ ^a^
**Bespoke support (i.e., email, phone, and face-to-face opportunities as needed)**								
Patients	✘	✓	✘	✘	✘	✘	✓ ^j^	✘
Patient groups	✘	✓	✓ ^a^	✘	✓ ^a^	✘	✓	✘
**Funding (i.e., to cover travel costs, etc.)**								
Patients	✘	✓	✘	✓ ^k^	✘	✘	✓ ^j^	✘
Patient groups	✘	✓	✘	✓ ^k^	✘	✘	✓ ^j^	✘

Notes: ✓ = materials found on the HTA website and/or in other identified sources. ✘ = materials not found on the HTA website and/or in other identified sources. (^a^) Source for this is not available in English. All sources are set out in ALAN’s comparative report [5]. N/A: not applicable, as IQWiG does not offer any direct early advice. Their experts are involved in G-BA’s early advice processes. (^b^) If no patient group exists, individual patients can be involved by exception. (^c^) In exceptional cases, the term patient is used in sources, but it is unclear if that is really patients or patient groups. (^d^) SMC feedback for PACE. (^e^) Feedback from CADTH, now CDA-AMC. (^f^) Feedback from NICE. (^g^) French patient organizations that submitted their written contribution cannot attend the CT’s meetings and therefore cannot vote. Only its twenty-nine permanent members, three of which are consumer and patient representatives, can vote, based on French sources. (^h^) Feedback from SMC. (^i^) IQWiG experts contribute regularly to the training sessions for patient representatives conducted by the G-BA. (^j^) SMC feedback, covering patient involvement in PACE. SMC also clarified that as they are part of Health Improvement Scotland, they follow the same policy for reimbursement of volunteer expenses. This means that patients and patient groups are all provided with details and support to claim expenses. (^k^) Source for this is direct exchange with an IQWiG representative.

## Abbreviations

The following abbreviations are used in this manuscript:

AEMPS	Spanish Agency for Medicines and Medical Devices
AIFA	Italian Medicines Agency
ALAN	Acute Leukemia Advocates Network
CADTH	Canadian Agency for Drugs and Technologies in Health
CDA-AMC	Canada’s Drug Agency
EMA	European Medicines Agency
EU	European Union
G-BA	Federal Joint Committee
HAS	French National Authority for Health
IQWiG	Institute for Quality and Efficiency in Health Care
JCA	Joint Clinical Assessment
JSC	Joint Scientific Consultation
HTA	Health Technology Assessment
PICO	Population, Intervention, Comparison, Outcome
NICE	National Institute for Health and Care Excellence
SMC	Scottish Medicines Consortium

## References

[1] O’Rourke B., Oortwijn W., Schuller T. (2020). The new definition of health technology assessment: A milestone in international collaboration. Int. J. Technol. Assess. Health Care.

[2] European Commission (EC) Health Technology Assessment, Overview. https://health.ec.europa.eu/health-technology-assessment/overview_en.

[3] European Commission (EC) (2021). Questions and Answers: Adoption of Regulation on Health Technology Assessment. https://ec.europa.eu/commission/presscorner/detail/en/qanda_21_6773.

[4] Wale J.L., Thomas S., Hamerlijnck D., Hollander R. (2021). Patients and public are important stakeholders in health technology assessment, but the level of involvement is low—A call to action. Res. Involv. Engagem..

[5] Acute Leukemia Advocates Network (ALAN) (2024). Patient Involvement in Health Technology Assessment of Medicines for the Purpose of Reimbursement: An Exploratory Comparative Report. https://acuteleuk.org/publications/.

[6] Office of Health Economics (OHE) (2024). Incorporating the Patient Voice in Health Technology Assessment. https://www.ohe.org/publications/patient-voice-in-hta.

[7] Haute Autorité de Santé (HAS) (2022). CT Commission de la Transparence. Rapport d’Activité. https://www.has-sante.fr/upload/docs/application/pdf/2023-06/rapport_dactivite_2022_de_la_ct.pdf.

[8] European Patient Forum (EPF) (2024). EPF Feedback on the Implementing Act on Joint Clinical Assessments of Medicinal Products. https://www.eu-patient.eu/news/latest-epf-news/2024/epf-feedback-on-the-implementing-act-on-joint-clinical-assessments-of-medicinal-products/.

[9] Cancer Patients Europe (CPE), European Federation of Allergy and Airways Diseases Patients’ Association (EFA), European Haemophilia Consortium (EHC), European Kidney Patients’ Federation (EKPF), European Multiple Sclerosis Platform (EMSP), European Patients Forum (EPF), European Pulmonary Fibrosis Federation (EU-PFF), France Assos Santé (FAS), Lymphoma Coalition, Myeloma Patients Europe (MPE) (2024). 10 Key Recommendations from Patient Organisations on Joint Clinical Assessments Under the EU HTA Regulation. https://www.eu-patient.eu/news/latest-epf-news/2024/10-key-recommendations-for-enhancing-joint-clinical-assessments-under-the-eu-hta-regulation/.

[10] European Patient Forum (EPF) (2024). EPF’s Feedback to the Implementing Act on HTA Cooperation with the EMA. https://www.eu-patient.eu/news/latest-epf-news/2024/epfs-feedback-to-the-implementing-act-on-hta-cooperation-with-the-ema2/.

